# Zoonotic Sporotrichosis Related to Cat Contact: First Case Report from Panama in Central America

**DOI:** 10.7759/cureus.2906

**Published:** 2018-07-01

**Authors:** Margarita E Rios, José Suarez, Jose Moreno, Jorge Vallee, Juan Pablo Moreno

**Affiliations:** 1 Tropical Medicine, Gorgas Institute of Health Studies, Panama, PAN; 2 Microbiology, Gorgas Institute of Health Studies, Panama, PAN; 3 School of Medicine, Latina University of Panama, Panama, PAN; 4 School of Medicine, Latina University of Panama, Panamá, PAN

**Keywords:** sporotrichosis, cats, zoonosis, central america, panama

## Abstract

A previously healthy 34-year-old man presented with a four-week-old upper arm injury that started with a cat scratch and progressed into painless erythematous nodular ascending lesions in the left upper limb. We made the diagnosis of sporotrichosis based on the epidemiological association of the contact with a cat and culture identification. Sporotrichosis is a mycosis implantation caused by the Sporothrix schenckii complex. We present the first Central American case of sporotrichosis associated with contact with a feline.

## Introduction

Sporotrichosis is a subcutaneous mycosis caused by the dimorphic fungus Sporothrix schenckii (S. schenckii), which is usually found in tropical and subtropical zones but has been found worldwide on occasion [1-2]. Typically, the infection develops after traumatic inoculation with contaminated soil, plants, and organic matter into the skin or mucosa. Infection may also occur via animal transmission (e.g., cat-to-cat or cat-to-dog) or zoonotic transmission (cat-to-human), usually via scratches or bites from infected cats. The zoonotic transmission has been reported from domestic animals like dogs and wild animals like armadillos but is most commonly associated with felines associated with patients with domestic jobs [1,3-4].

The number of reported cases of zoonotic infections from feline-related sporotrichosis is increasing. Zoonotic cases have been reported in the United States (US), India, Malaysia, Argentina, and Mexico, but no cases of zoonotic transmission have been reported from the Central American region until this one [4-5].

According to Ministerio de Salud: Resolución No. 2283, “due to the high incidence of feline sporotrichosis, Rio de Janeiro is presently considered hyperendemic for cat-associated sporotrichosis” [6] and has the largest absolute number with 4669 cases and an overwhelming prevalence of Sporothrix brasiliensis during epizootic cat-related outbreaks. Cats represent a significant zoonotic potential given their lesions are rich in fungus, and they are usually close to humans [1,4,6].

Panama is considered a leishmaniasis hyperendemic area and has a population at 75% risk for contracting cutaneous leishmaniasis [7]. In Panama, there are 40.2 cases of leishmaniasis per 10,000 inhabitants [7-8].

The clinical presentation of sporotrichoid leishmaniasis has been observed in America and in our clinical practice. Differentiating cutaneous leishmania from sporotrichosis is a clinical diagnostic challenge given the similarity of the lesions and the epidemiologic antecedent of inoculation of the fungus through thorns, splinters, and small scratches received during leisure and occupational activities, such as floriculture, horticulture, gardening, fishing, hunting, farming and cattle raising, mining, and wood exploration [9-10].

Our case presents an atypical zoonotic transmission of sporotrichosis and represents the first such reported case in Panama.

## Case presentation

A previously healthy 34-year-old man from La Espiga, Chorrera district, Province of West Panama, presented to our institution with ipsilateral, painless, erythematous, nodular ascending lesions in his left hand, forearm, arm, and axillary region. The lesions followed a lymphangitic path. The patient reported that one month before presentation, the lesions started as a cat scratch on the third finger of his left hand. The lesions were not associated with fever or signs of infection. The patient is a laundryman who is in regular contact with eight cats, and he has no history of trauma from the manipulation of plants, leisure, or occupational activities. Before presenting to our clinic, he went to a health center where he was administered topical silver sulfadiazine, but the condition did not improve.

**Figure 1:**
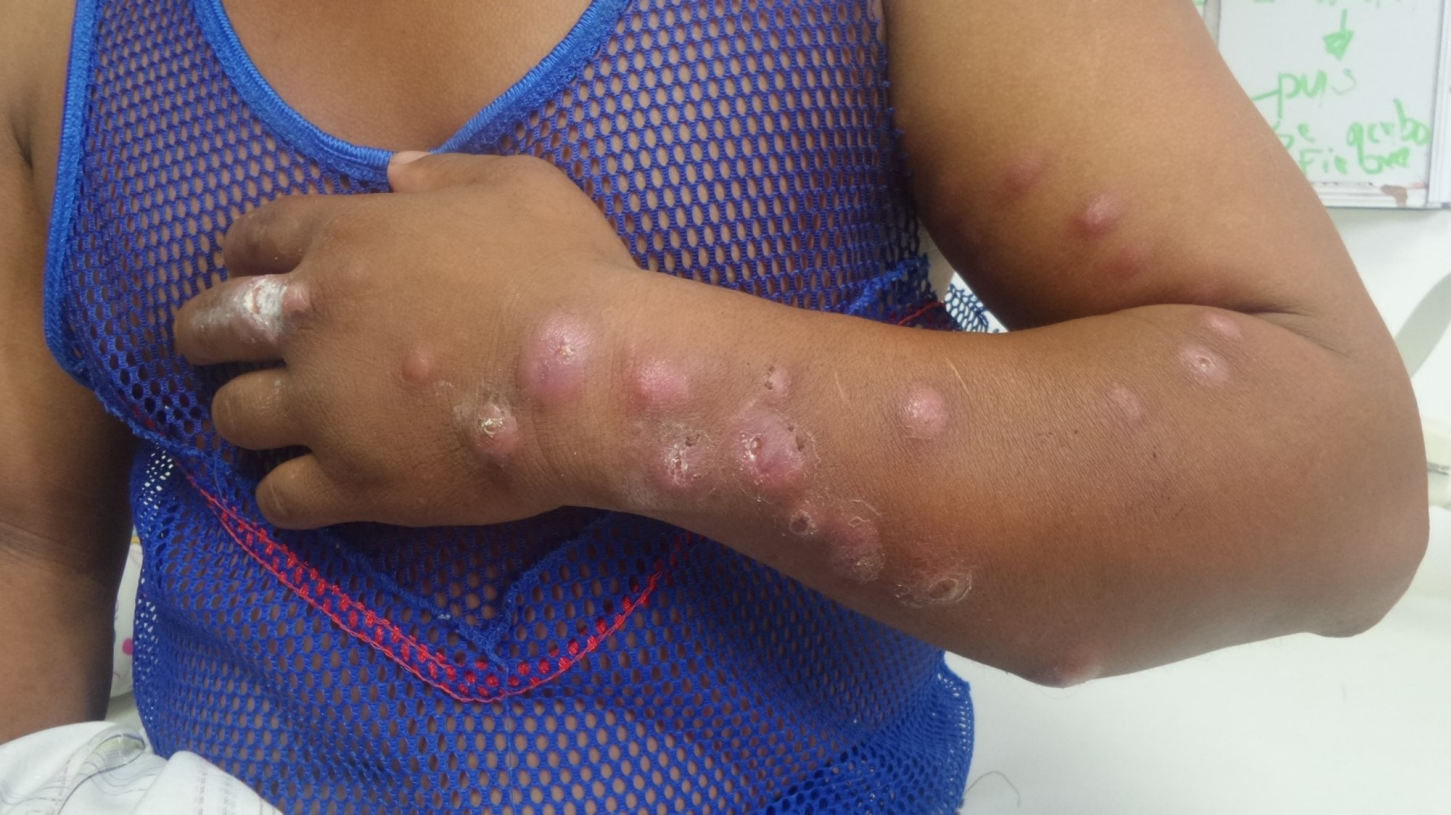
Patient with cutaneous sporotrichosis

On physical examination, we noted a nodular, erythematous, ulcerated lesion on the middle finger of his left hand with additional nodular, erythematous lesions that were not painful on palpation and had no differences in temperature. Some of those lesions were ulcerated. We found three similar lesions on the dorsum of his left hand. On the patient’s forearm, we found nine lesions on the posterior forearm, four lesions on the anterior forearm, and one on the medial forearm. On his arm, we found three lesions in the anterior area, seven in the medial area, and four in the lateral area (Figure 1). We noted one nodular lesion in the patient’s axillary area.

The 72-hour Montenegro skin test yielded a negative result. On the day-10 follow-up visit, we scraped the lesion for polymerase chain reaction (PCR) testing, culture and smear for Leishmania, and an acid-fast bacillus (AFB, also known as bacillus acido-alcoolo résistants (BAAR)) smear for cutaneous tuberculosis. The result of the AFB test was negative.

We prepared Saboraud cultures to rule out sporotrichosis, placing the samples on two slides for visualization. On the first slide, we added a drop of 20% potassium hydroxide, in the other, a drop of lactophenol blue. After 10 minutes, we observed the slides at 400x and 1000x magnification for fungal structures. The positive sample was seeded on Saboraud agar and Micosel (CONDA Pronadisa, Spain).

After 15 days of incubation with notorious fungal growth, we incubated a culture. A 1-cm^2^ segment of sterile Saboraud agar was taken with a sterile scalpel and placed on a clean slide. This segment was then inoculated with a needle loaded with the fungal culture. A sterile slide was mounted and placed in a Petri dish with a paper towel wetted with sterile water as a wet chamber. The culture was incubated at room temperature until growth was observed on the coverslip, which was then carefully peeled off to mount it on a slide cleaned with 70% ethanol with a drop of lactophenol blue for microscopic observation for structures such as conidia, hyphae, or spores indicative of the S. schenckii complex.

The patient was treated with itraconazole 100 mg orally each day for 90 days and recovered with a full resolution of the lesions.

## Discussion

The diagnosis of sporotrichosis in endemic areas consists of an epidemiological diagnosis and a history of accident, trauma, puncture, or contact with plants and vegetation, as well as any exposure risk associated with the patient’s occupation [1,6]. Additionally, leishmaniasis coexists in many countries endemic to sporotrichosis, and, from a clinical perspective, leishmaniasis presents as a clinical variant of sporotrichoid leishmaniasis, which is itself often clinically indistinguishable from lesions caused by a Sporothrix schenckii infection [7,9]. In some cases, the differential diagnosis is evident because of a history of a bite or accidental puncture with a plant or, failing that, a risk of occupational exposure. However, sometimes, the epidemiological background and work history are not helpful. In those instances, arriving at the differential diagnosis should be a systematic process to distinguish between sporotrichosis and leishmaniasis [1,6,10]. The zoonotic transmission of sporotrichosis is atypical and infrequent, so an accurate history is vital when assessing a patient’s clinical background, and patients should be asked about recent contact with animals such as cats or armadillos.

In any consultation of traveler's medicine (i.e., medical conditions associated with traveling to areas endemic for certain diseases), dermatology, or tropical medicine, samples should be collected with the intent to rule out both leishmaniasis and sporotrichosis. In our case, according to the Republic of Panama guidelines for leishmaniasis, we performed PCR, smear, and culture, and searched for mycotic isolation to arrive at the diagnosis of sporotrichosis [5,8,11].

Cutaneous leishmaniasis and sporotrichosis are managed differently. Cutaneous leishmaniasis is treated with pentavalent antimonials as the first-line treatment; sporotrichosis is treated with imidazole antifungals such as itraconazole. A correct differential diagnosis is important for implementing an adequate therapeutic approach [5,8]. Our case illustrates this concept given the patient’s history of close contact with cats and the presence of a scratch as an initial injury. The zoonotic transmission was important to the etiopathogenesis of the patient’s condition. Few such cases have been reported in the world, and this case represents the first reported case in Panama [1-3,12]. While the classic presentation of sporotrichosis with epidemiological antecedents is important, zoonotic transmission has not been extensively studied or reported, and the number of cases related to zoonotic transmission may increase [2-3,12].

## Conclusions

Sporotrichosis is traditionally associated with occupational exposure in patients active in farming, gardening, or otherwise manipulating plants. Zoonotic transmissions are rarely considered when diagnosing this entity, and evidence suggestive of sporotrichosis is usually revealed while taking the patient’s history where accidental exposure to plants is disclosed. Our case represents the first report of the zoonotic transmission of sporotrichosis in the Central America region. Other researchers and healthcare providers in Central America should consider zoonotic transmission within their diagnostic algorithms, especially when evaluating patients with characteristics similar to those reported in this case who may have been in contact with animals such as cats or armadillos.
